# Long non-coding RNA LINC01503 promotes the progression of hepatocellular carcinoma via activating MAPK/ERK pathway

**DOI:** 10.7150/ijms.45256

**Published:** 2020-05-18

**Authors:** Mu-Ru Wang, Dan Fang, Mu-Ping Di, Jia-Lun Guan, Ge Wang, Lian Liu, Jia-Qi Sheng, De-An Tian, Pei-Yuan Li

**Affiliations:** 1 Division of Gastroenterology, Tongji Hospital, Tongji Medical College, Huazhong University of Science and Technology, Wuhan 430030, China; 2 Sun Yat-sen University Cancer Center, State Key Laboratory of Oncology in South China, Collaborative Innovation Center for Cancer Medicine, Guangzhou 510060, China; 3 Department of Gastroenterology, Beilun Branch of the First Affiliated Hospital, College of Medicine, Zhejiang University, Ningbo 315800, China

**Keywords:** hepatocellular carcinoma, LINC01503, MAPK/ERK pathway, proliferation, apoptosis

## Abstract

**Background**: Increasing evidence has implicated that lncRNAs (long non-coding RNAs) play significant roles in carcinogenesis and progression of HCC (hepatocellular carcinoma). LINC01503 is a new lncRNA related to several tumors. Nonetheless, its role in HCC still remains unclear.

**Methods**: The expression levels of LINC01503 in HCC, normal liver tissues as well as HCC cell lines were evaluated by TCGA (The Cancer Genome Atlas) and real-time PCR assay, respectively. The relationship between LINC01503 levels and the prognosis of patients with HCC was evaluated using Kaplan‐Meier survival analysis. Then the potential biological functions and pathways related to LINC01503 were investigated by GO (Gene Ontology) analysis and KEGG (Kyoto Encyclopedia of Genes and Genomes) analysis, and GSEA v4.0.1 software was employed. Furthermore, the influence of LINC01503 on the proliferation and apoptosis of HCC cells was confirmed using CCK8 assay, flow cytometry, and clone formation assay in cell experiments. Also the pro-tumor effect of LINC01503 was verified by mice xenograft experiment *in vivo*. In addition, the functional pathway of LINC01503 was proved by western blot and rescue experiments.

**Results**: LINC01503 was highly expressed in HCC and positively correlated with large tumor size, high tumor grade, advanced tumor stage, and poor prognosis of HCC patients. Silencing LINC01503 with shRNA significantly restrained the proliferation of MHCC-97H HCC cells and strengthened the apoptosis, while up-regulation of LINC01503 in Huh7 HCC cells contributed to the contrary effects. Besides, LINC01503 promoted tumor growth of nude mice transplanted with liver cancer cells. Mechanistically, MAPK/ERK signaling pathway was activated by LINC01503, inhibition of which could alleviate the pro-tumor effect of LINC01503, consistent with the forecast of GSEA (Gene Set Enrichment Analysis).

**Conclusion**: LINC01503 is highly expressed in HCC and promotes the progression of HCC via MAPK/ERK pathway, which maybe a new potential biomarker and therapeutic target for HCC.

## Introduction

HCC (hepatocellular carcinoma) ranks as the 2^nd^ major reason for deaths related to tumor in the world, which has extremely high mortality with around 800,000 patients newly diagnosed each year 1, 2. Although there are several treatment modalities for HCC, such as trans-catheter arterial chemoembolization (TACE), liver transplantation, ablation, surgical excision, and targeted molecular chemotherapy, the 5-year survival rate in patients with HCC still remains poor because of the intrahepatic metastasis and frequent recurrence 3. Hence, it is imperative to elucidate the occurrence and development mechanism of HCC and discover novel therapeutic targets to lessen the mortality of HCC patients.

lncRNAs (long noncoding RNAs) belong to the ncRNA family whose length ranges from 200 to over one hundred thousand nucleotides, lacking of protein-coding ability. LncRNAs participate in the regulation of genes expression via multitudinous mechanisms, which have been confirmed to function in various biological activities, including apoptosis, metastasis, cell proliferation, tumorigenesis and differentiation 4, 5. So far, it has been demonstrated that lncRNAs participate in the progression of a variety of diseases. Recent studies revealed that lots of non-coding transcripts were dysregulated in various cancers and played significant roles in the carcinogenesis including HCC 6, 7. Therefore, lncRNAs might become potential therapeutic targets of hepatocellular carcinoma.

Recently, it has been shown that LINC01503 (long intergenic non-protein coding RNA 01503) is related to the progression of ESCC (esophageal squamous cell carcinoma). Overexpressed LINC01503 facilitates cell migration, invasion, and proliferation 8. In addition, LINC01503 has also been proved to play oncogenic roles in several cancer types, including colorectal cancer, glioma, and cholangiocarcinoma 9-11. Nonetheless, the expressing level and function of LINC01503 in HCC still remain unclarified. Therefore, this research was designed to characterize the function of LINC01503 in HCC.

In our research, according to the analysis of TCGA (The Cancer Genome Atlas) data, LINC01503 was highly expressed in hepatocellular carcinoma (HCC) and positively correlated with large tumor size, high tumor grade, advanced tumor stage, and poor prognosis of HCC patients. Moreover, knockdown of LINC01503 significantly suppressed proliferation and promoted apoptosis of HCC cells, while overexpression of LINC01503 had the contrary adverse effects *in vitro* and *in vivo*. Mechanistically, LINC01503 accelerated proliferation and prohibited the apoptosis process via the MAPK (mitogen-activated protein kinase) / ERK (extracellular signal-regulated kinase) pathway, a crucial pathway for human cancer 12-14.

## Materials and Methods

### TCGA (The Cancer Genome Atlas) cohort and GSEA (Gene Set Enrichment Analysis)

The gene expression profiles and clinical data of HCC patients were obtained from the TCGA data portal (https://www.cancer.gov/tcga/) in April 2019. TCGA data included 50 normal liver tissues and 374 HCC tissues (Table 1). HCC samples were divided into low LINC01503 expression group or high LINC01503 expression group, and the median value was set as cutoff. Next, GSEA v4.0.1 was applied to perform GO analysis and KEGG analysis to investigate the biological function and pathway involved in HCC pathogenesis through LINC01503.

### HCC samples

Pair-matched tumor tissues and adjacent non-tumorous tissues from 35 patients undergoing resection of HCC were obtained from Tongji Hospital, Huazhong University of Science and Technology in 2016. Informed consent was obtained from all of the patients. The study was performed according to the guidelines of the Ethics Committee of the Tongji Hospital and approved in accord with the ethical standards of World Medical Association Declaration of Helsinki.

### Cell culture

The cell lines of human HCC including Huh7, MHCC-97H, Hep-G2, Hep-3B, HCCLM3, MHCC97-L and L02 liver cells were kept in the Institute of Liver Diseases (Tongji Hospital, Wuhan, China) and cultured after resuscitation using DMEM supplemented with fetal bovine serum (10%, Gibco, Grand Island, NY, America) in a 37℃ incubator with 5% CO2.

### Plasmids, virus, and transfection

Transfection of the plasmid of luciferase reporter was carried out using the reagent of Lipofectamine 3000 (Invitrogen, Carlsbad, America) following the instructions from the manufacturer. For overexpression of LINC01503, human full-length LINC01503 gene was acquired using PCR assay and inserted into the pMSCV plasmid. To downregulate LINC01503, shRNA sequence which targeted LINC01503 was inserted into the pSuper-puro plasmid. The transfection assay was performed using Lipofectamine 3000 (Invitrogen, Carlsbad, America). The target cells were infected using retroviruses generated by pMSCV-puro-LINC01503 and pSuper-puro-LINC01503-shRNA for 72 h. Puromycin (0.6 μg/mL) was employed to screen the stable cell lines for 9 days.

### Extraction of RNA and real-time PCR assay

Trizol reagent (Invitrogen, Carlsbad, America) was used to extract total RNA, and the manufacturer manual was followed. The complementary DNA which was reversely transcribed was synthesized by Prime Script RT Reagent Kit (Takara, Tokyo, Japan). Real-time PCR assay was carried out as what was mentioned previously 15. The 2-ΔΔCt method was applied to determine the difference between multiple samples. LINC01503 primer sequence: sense strand, 5'-TCTTCGTGTTCATCATCAGTCCC-3'; antisense strand, 5'-CTGAAAGAAACTCATTGCATCGTG-3'.

### Histology

After the mice were sacrificed, the tumor samples were obtained and fixed with paraformaldehyde (4%), followed by paraffin embedding. The sections were prepared and stained by H&E. Furthermore, tumor tissues were applied to conduct immunohistochemistry assay for Ki67. The procedures were carried out as what were described previously 16.

### Western blot analysis

Western blot assay was conducted as mentioned previously 16. The primary antibodies were listed below: anti-BCL2 apoptosis regulator (BCL2) antibody (Proteintech, 12789-1-AP, 1:1000), anti-BCL2 associated X, apoptosis regulator (BAX) antibody (Proteintech, 50599-2-Ig, 1:1000), anti-ERK1/2 antibody (CST, 4695, 1:1000), anti-phosphoERK1/ 2 (p‐ERK1/2) antibody (CST, 4370, 1:1000), anti-JNK antibody (CST, 9252, 1:1000), anti-phosphoJNK (p‐JNK) antibody (CST, 9251, 1:1000), anti-P38 antibody (CST, 8690, 1:1000), anti-phosphoP38(p-P38) antibody (CST, 4511, 1:1000) and anti-α-tubulin antibody (CST, 2125, 1:1000). Anti-rabbit IgG (Jackson ImmunoResearch Laboratories, America, 1:2000) was used as the secondary antibody. The protein bands were scanned and analyzed by using ImageJ software (NIH, America).

### CCK8, clone formation, and cell apoptosis experiments

Cell proliferation ability was measured by the Cell Counting Kit-8 (CCK-8, Promotor, China) following the instructions. After addition of CCK-8 reagent into the 96-well plates, the cells were cultured for two hours. The absorbance at 450 nm (OD450) was recorded. Clone formation assay was used to evaluate the clone formation capability of HCC cells. Cells (1 × 10^3^/well) were seeded in 6-well plates. Afterwards, the cells were maintained in DMEM (Promoter, Wuhan, China) which was added with fetal bovine serum (10%, Gibco, Grand Island, NY, America). After 2 weeks, the colonies were fixed by using paraformaldehyde (4%, Servicebio, Wuhan, China) in a 37 °C incubator for four hours, and then stained by crystal violet (0.5%, Promoter, Wuhan, China) for 2 h at 37 °C. The colony number was counted under a light microscope. Annexin V-FITC / propidium iodide (annxinV/PI) apoptosis detecting kits (Nanjing KeyGen Biotech, Nanjing, China) were used to determine the cell apoptosis, and the cells were stained. Flow cytometer (FACSCalibur, BD Biosciences) was carried out to determine the apoptosis ability and the results were analyzed with CellQuest software. SCH772984 (1μM, a highly selective and ATP-competitive ERK inhibitor, Medchem Express, America) was used to inhibit ERK1/2 in this study.

### In vivo tumor xenograft model

BALB/c nude mice (male; age, 3-4 weeks; body weight, 12-14g) were purchased from Beijing HFK Bioscience. Mice were maintained in a pathogen-free environment (relative humidity, 60% ± 5%; temperature, 22 ± 2°C) with a light / darkness cycle of 12 h/12h. The mice were allowed free access to common chow diet and water in the Center of Animals in Tongji Hospital. All in-vivo experiments were performed in adherence to the Guide for the Care and Use of Laboratory Animals of Tongji Medical College. HCC cells (5×10^6^) were suspended in PBS (0.1 mL) and injected subcutaneously into the mice from the flank of both sides at the dorsal region to establish a hepatoma xenograft model. Tumor size was monitored by measuring the length (L) and width (W) with digital caliper, and tumor volume (V) was calculated based on the equation V = 1/2 × L × W^2^. On the 21^st^ day, the mice were injected with luciferin (150 mg/kg) in the intraperitoneal cavity. The tumors were observed using a Lago X imaging system (SI Imaging) about 5 minutes post luciferin injection, excised and weighed.

### Statistical analyses

All analysis of the statistics was carried out by R (v.3.4.3) or SPSS (version 25, IBM Corp, Armonk, NY, America). The correlation between LINC01503 and clinicopathological features was assessed by the logistic regression and Wilcoxon signed-rank test. The Kaplan-Meier method or Cox regression was used to evaluate the relationship between Clinicopathologic characteristics and overall survival (OS) in TCGA patients. Univariate and multivariate Cox regression analysis were used to identify whether the value of LINC01503 expression levels could be used as an independent risky factor, factors with *P* < 0.05 in univariate regression analysis were entered in the multivariate regression models. Student's t-test was utilized to compare means between two groups, and one-way analysis of variance was used for multiple groups. All statistical analyses were two-sided. *P*<0.05 indicated significant difference.

## Results

### The expression of LINC01503 is up-regulated in HCC and correlates with poor clinical outcomes

**Figure 1:**
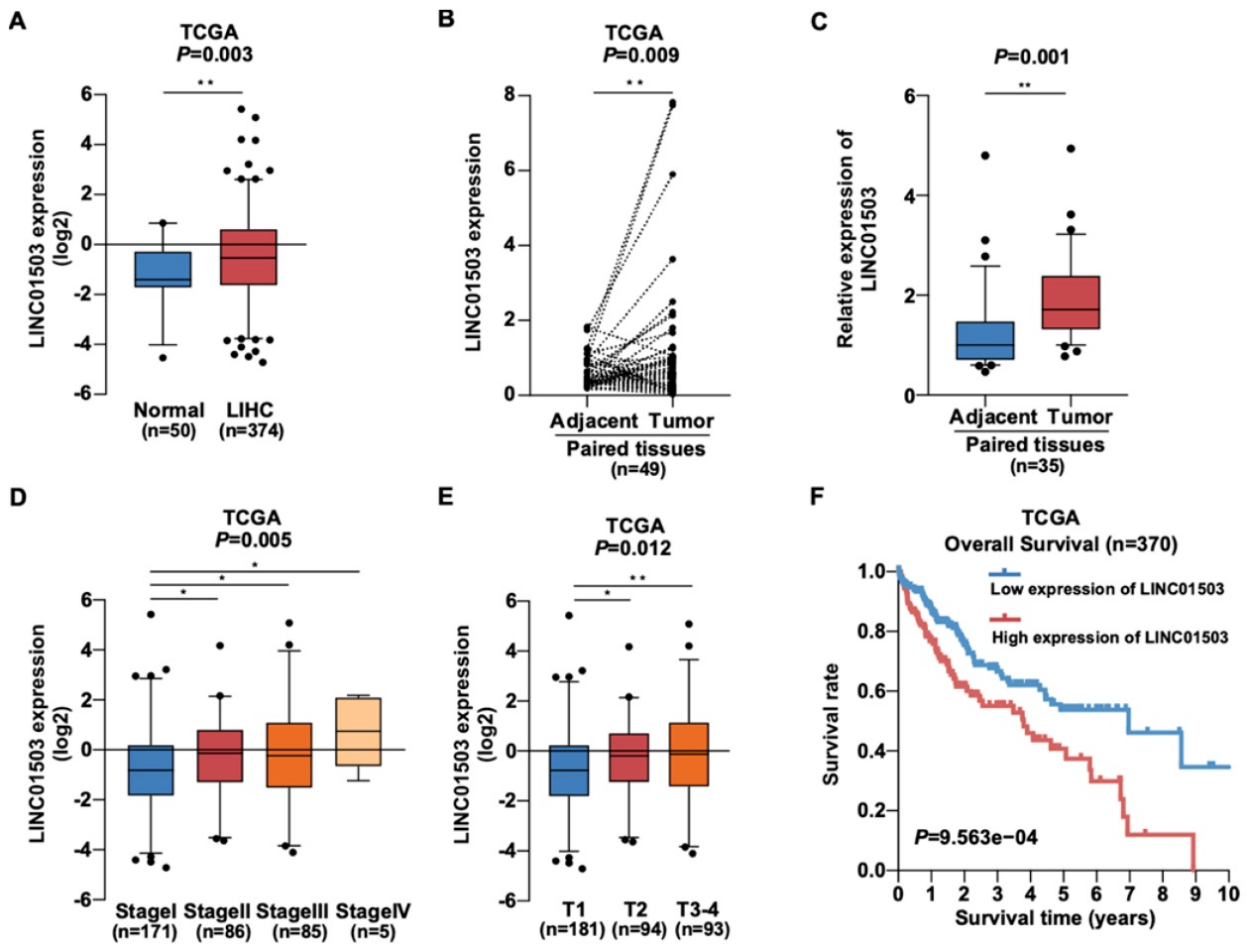
** The expression of LINC01503 is up-regulated in HCC and correlates with poor clinical outcomes. (A)** The expression level of LINC01503 in normal liver samples and HCC samples, as indicated by TCGA LIHC dataset including 374 HCC samples and 50 normal liver samples.** (B)** LINC01503 in HCC tissues and paired adjacent tissues according to the TCGA LIHC dataset. **(C)** Analysis of LINC01503 expression levels in HCC tissues and normal liver samples collected in our hospital using real-time PCR.** (D and E)** The relationship between LINC01503 expression and stage, tumor size (T) in TCGA LIHC data. **(F)** Effects of LINC01503 levels on the overall survival rate of HCC patients in the TCGA cohort. *, *P*<0.05, **, *P*<0.01. TCGA, The Cancer Genome Atlas; LIHC, liver hepatocellular carcinoma; HCC, hepatocellular carcinoma.

To learn about the expression of LINC01503 in HCC, we downloaded gene expression profiles and clinical information of 50 normal liver samples and 374 HCC samples from TCGA data portal in April 2019. The clinical data of 374 HCC samples is shown in Table 1. According to the analysis of TCGA HCC data, the LINC01503 expression was dramatically increased in HCC samples in comparison with normal liver tissues (*P*=0.003) (Figure 1A). Similarly, the LINC01503 expression was substantially upregulated in HCC tissues in comparison with the adjacent non-tumorous tissues (P=0.009) (Figure 1B). Furthermore, the results of LINC01503 expressions were further verified in 35 HCC samples and adjacent non-tumorous tissues collected in our hospital by real-time PCR analysis (P=0.001) (Figure 1C). These results indicated that LINC01503 levels are dramatically increased in HCC.

Then we wanted to know whether LINC01503 is related with HCC patients' clinicopathologic characteristics. Interestingly, TCGA data showed that high LINC01503 level correlated with higher stage (*P*=0.005) and larger tumor size (T) (*P*=0.012) (Figure 1D, E). The results of logistic regression analysis suggested that the LINC01503 level as one of the categorical dependent variables (according to the median value of expression) was related to pathological features (Table 2). The LINC01503 levels were correlated closely with grade (OR = 4.86 for IV *vs*. I, *P*=0.029), stage (OR= 1.82 for II *vs*. I,* P*=0.025; OR= 1.78 for III *vs*. I,* P*=0.031), and T classification (OR= 1.65 for T2~4 *vs*. T1,* P*=0.017) (Table 2). The results above indicated that HCC with a high LINC01503 level tended to proceed to a worsen stage, grade and T classification compared with that with a low LINC01503 level.

Additionally, Kaplan-Meier survival analysis demonstrated that HCC patients with high LINC01503 expression exhibited significantly worse overall survival than those with low LINC01503 expression (*P*=9.563e-04) as shown in Figure 1F. Multivariate and univariate Cox regression analyses indicated that LINC01503 levels could be used as an independent risky factor of overall survival among patients with HCC (HR=1.26, *P*=0.046) (Table 3).

### The expression of LINC01503 is relatively high in HCC cell lines

**Figure 2:**
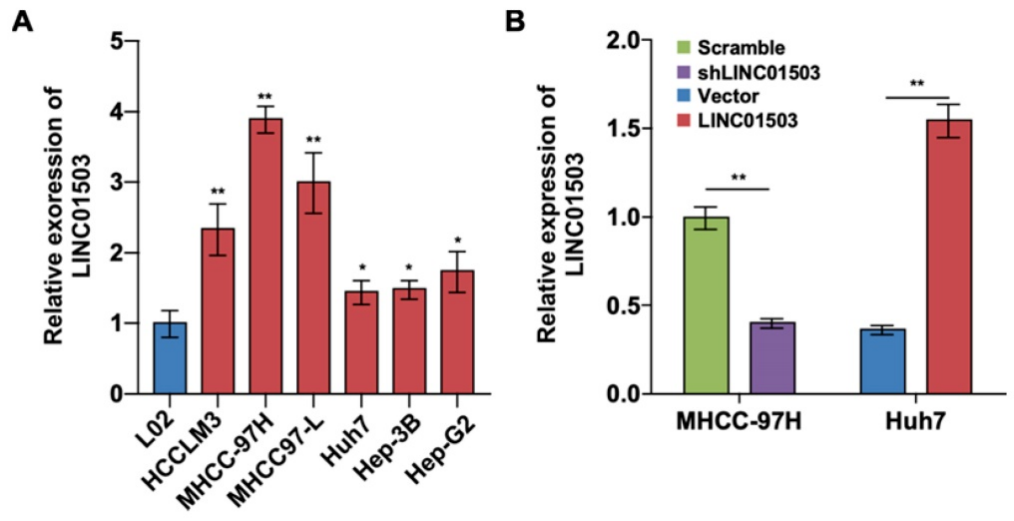
** The LINC01503 expression and regulation in HCC cell lines. (A)** Relative expression levels of LINC01503 in HCC cell lines (HCCLM3, MHCC-97H, MHCC97-L, Huh7, Hep-3B and Hep-G2) and L02 liver cells measured by using real-time PCR. **(B)** The regulation of LINC01503 levels in HCC cells. MHCC-97H cells were transfected by shRNA targeting LINC01503 (shLINC01503), and Huh7 cells were transfected by pMSCV plasmids encoding LINC01503 (LINC01503). Cells were transfected by scrambled shRNA (scramble) or pMSCV (vector) and served as negative control, respectively. LINC01503 was measured by real-time PCR. Data were shown as mean ± SD, n = 3. *, *P*<0.05, **, *P*<0.01. shRNA, short hairpin RNA.

To further investigate the expression and effect of LINC01503 in HCC cells, we detected the expression levels of LINC01503 by using real-time PCR assay. The results showed that LINC01503 expression levels were upregulated in HCC cell lines (especially in MHCC-97H cell lines) compared with normal liver cells (L02 cell) (*P*<0.05) (Figure 2A). The endogenic LINC01503 expression was downregulated by shRNA in MHCC-97H cell line, which had a high basal expression of LINC01503. Meanwhile, LINC01503 was overexpressed in Huh7 cell line with pMSCV plasmids, which had a relatively low basal expression of LINC01503 (*P*<0.01) (Figure 2B).

### LINC01503 promotes cell proliferation and clone formation in HCC cells

**Figure 3:**
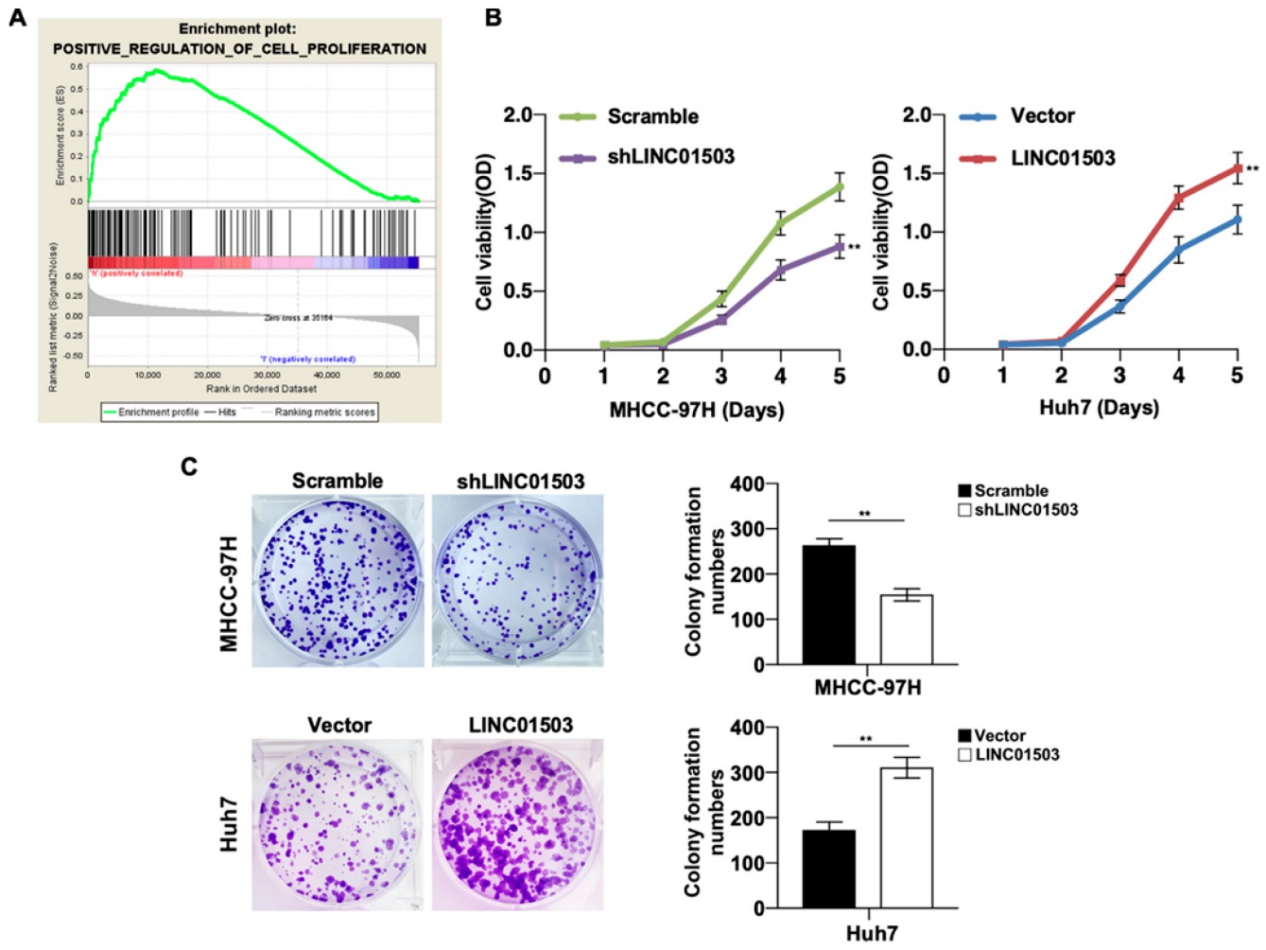
** LINC01503 promoted HCC cells proliferation. (A)** The correlation between LINC01503 expression levels and proliferation in HCC was indicated by GO analysis. **(B)** Cell proliferation was assayed in MHCC-97H-scramble, MHCC-97H-shLINC01503, Huh7-vector, and Huh7-LINC01503 cells using CCK8. **(C)** The clone formation was detected by crystal violet staining. Data were shown as mean ± SD, n = 3. **, *P*<0.01. GSEA, Gene Set Enrichment Analysis.

Since LINC01503 is highly expressed in HCC tissues and cell lines, we then wanted to study its potential biological functions involved in HCC pathogenesis. GSEA v4.0.1 was applied to perform GO analysis among the HCC samples in TCGA database. It demonstrated that high expression of LINC01503 was mainly involved in positive regulation of cell proliferation (Table 4) (Figure 3A). To confirm this, we performed CCK8 assay. As expected, cell proliferation ability was decreased when LINC01503 was silenced in MHCC-97H cell line but significantly enhanced when LINC01503 was upregulated in Huh7 cell line (*P* < 0.01) (Figure 3B). Consistently, similar results were found in the clone formation abilities of HCC cells (*P* < 0.01) (Figure 3C). Taken together, LINC01503 could positively regulate cell proliferation and clone formation in HCC cells.

### LINC01503 inhibits cell apoptosis in HCC cells

**Figure 4:**
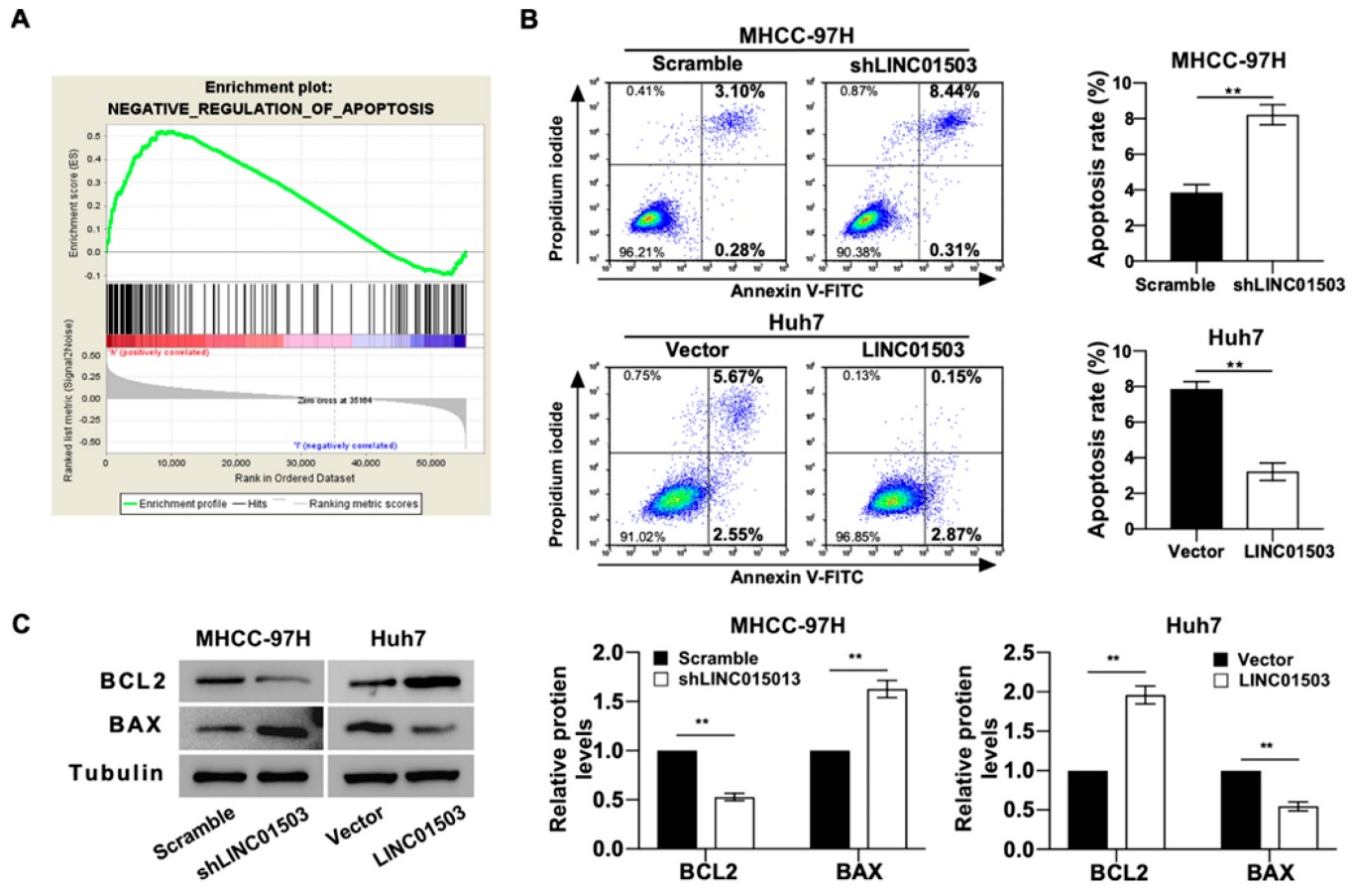
** LINC01503 inhibits HCC cells apoptosis. (A)** The correlation between LINC01503 expression levels and apoptosis in HCC was indicated by GO analysis. **(B)** Apoptosis of MHCC-97H and Huh7 cells assessed by flow cytometry. **(C)** The expression of BAX and BCL2, apoptosis-related proteins, was measured by western blot. Data were shown as mean ± SD, n = 3. **, *P*<0.01. GSEA, Gene Set Enrichment Analysis; BCL2, BCL2, apoptosis regulator; BAX, BCL2 associated X, apoptosis regulator.

GO analysis also indicated that LINC01503 expression levels were significantly correlated with the negative regulation of apoptosis (Table 4) (Figure 4A). We also tested this by apoptosis assays *in vitro*. Flow cytometry assay implied that silence of LINC01503 promoted apoptosis of MHCC-97H cells. On the contrary, overexpression of LINC01503 inhibited cell apoptosis in Huh7 cell line (*P*<0.01) (Figure 4B). Similar results were found by western blot assay, which demonstrated a decreased BCL2 expression, an anti-apoptotic protein, and an increased BAX level, the pro-apoptotic protein, in LINC01503 silence group compared to the control. There was an increased BCL2 expression and decreased BAX expression in the LINC01503 upregulation group in comparison with the control (*P*<0.01) (Figure 4C). These results implied that LINC01503 inhibited apoptosis of HCC cells.

### LINC01503 promotes xenograft tumor growth in vivo

**Figure 5:**
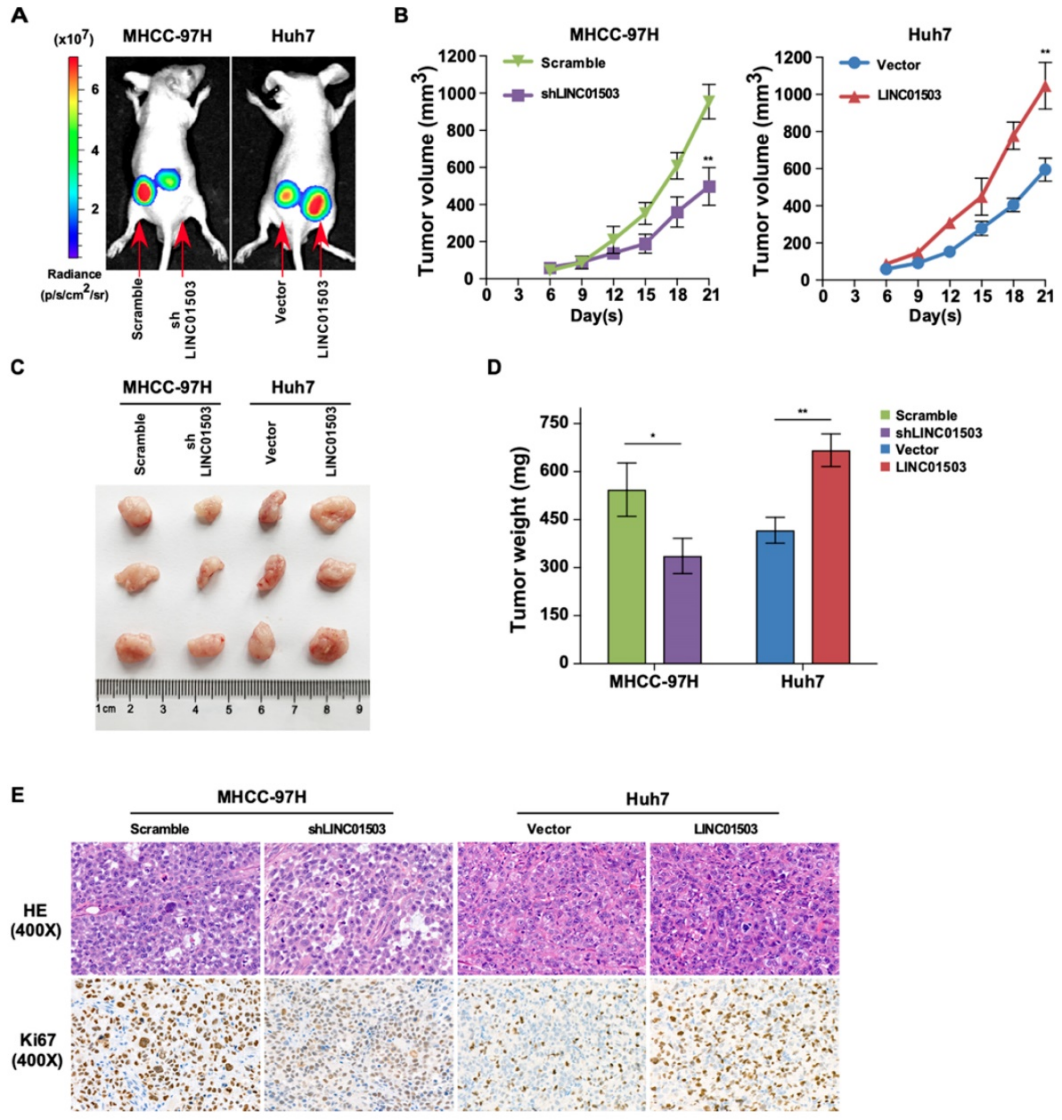
** LINC01503 promotes tumor growth of implanted HCC cells *in vivo*. (A)** Representative luciferase signal images of the tumor-bearing mice on the 21^st^ day post-implantation. **(B)** The growth curves of xenografts via measuring the volume of the tumor every 3 days after implantation. **(C)** Images of dissected tumors on the 21^st^ day post-implantation. **(D)** Tumor weight analysis. **(E)** Representative images of H&E (400×) and Ki67 IHC staining (400×) for tumor tissues. *, *P*<0.05, **, *P*<0.01.

Since LINC01503 could promote proliferation and inhibit apoptosis of HCC cells *in vitro*, we wanted to learn whether it could play a consistent role on tumor growth *in vivo*. Xenograft mouse models were established by subcutaneously inoculating control cells and cells with upregulated or silenced LINC01503 respectively into the left and right flank at dorsal region. After 21 days, Lago X imagining system demonstrated that the group with silencing of LINC01503 showed weaker fluorescence signals compared to the control. Conversely, LINC01503 overexpression group showed stronger fluorescence signals compared with the control group, as shown in Figure 5A. Consistent with the above data, knockdown of LINC01503 in MHCC-97H cells ameliorated the tumor growth rate compared with the control cells, whereas LINC01503 upregulation in Huh7 cells showed an opposite effect (*P*<0.01) (Figure 5B).

On the 21^st^ day following inoculation, the tumors were excised, photographed, and weighed after the mice were sacrificed, as shown in Figure 5C. LINC01503 knockdown in MHCC-97H cell line apparently decreased the tumor weight compared with the control group while LINC01503 overexpression in Huh7 cell line showed converse effects (*P*<0.05) (Figure 5D). Moreover, the protein expression of Ki67 in tumors was determined by immunohistochemistry. Compared with the control group, MHCC-97H derived tumors which were transfected by shLINC01503 exhibited lower levels of Ki67. On the contrary, more Ki67 positive signals were observed in LINC01503-overexpressing tumors implanted by Huh7 compared with the control group (Figure 5E). These data implied that LINC01503 could promote tumor growth of HCC* in vivo.*

### LINC01503 affected the biological behaviors of HCC cells through MAPK/ERK pathway

**Figure 6:**
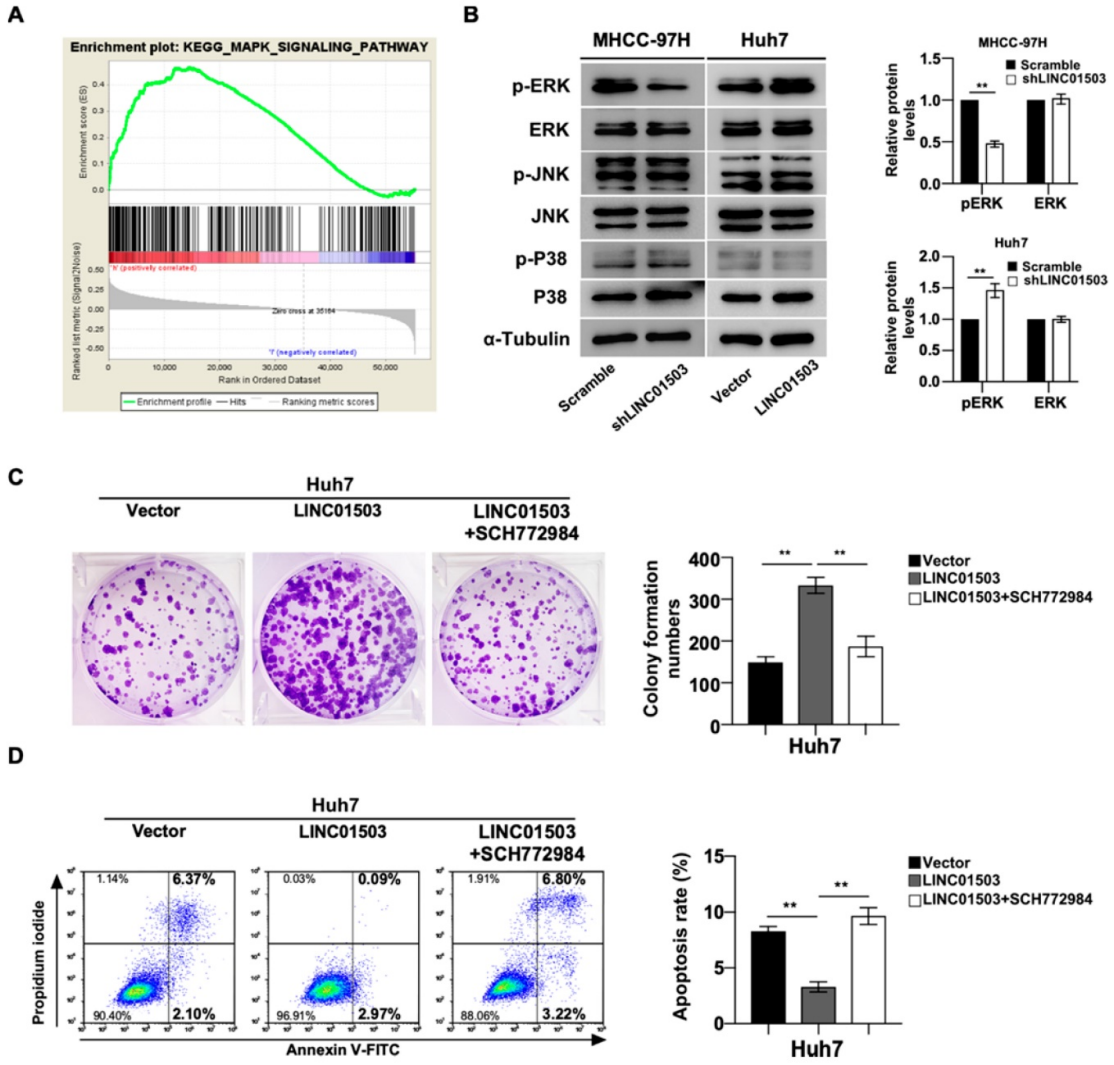
** LINC01503 affected HCC progression through MAPK/ERK pathway. (A)** KEGG analysis showed significant enrichment of MAPK signaling activation along with LINC01503 expression levels. **(B)** Western blot analysis for P38, p-P38, JNK, p-JNK, ERK1/2 and p-ERK1/2 in cell lines with altered LINC01503 expression levels. **(C, D)** Clone formation was assayed by crystal violet staining and apoptosis was assayed by flow cytometry in Huh7-LINC01503 cells with or without ERK inhibitor SCH772984 (1μM). Data were shown as mean ± SD, **, *P*<0.01. MAPK, mitogen-activated protein kinase; ERK, extracellular signal-regulated kinase; JNK, c-jun kinase; P38, p38 kinase; SCH772984, ERK inhibitor; GSEA, Gene Set Enrichment Analysis.

As shown above, LINC01503 displayed marked pro-tumor effects* in vitro* and *in vivo*, the mainly related mechanisms were further explored. KEGG analysis showed MAPK signaling pathway was remarkably enriched in HCC samples with high expression of LINC01503 (Table 4) (Figure 6A). Based on this clue, we validated whether LINC01503 affected the biological behaviors of HCC cells via MAPK pathway. The western blot analysis demonstrated there was decreased phosphorylation of ERK in MHCC-97H cells in the LINC01503 silence group compared to the control. On the contrary, overexpression of LINC01503 increased the phosphorylation of ERK in Huh7 cell line (*P*<0.01) (Figure. 6B). Next, we performed rescue assays in Huh7 cells using the specific inhibitor of ERK (SCH772984). The increased clone formation capability and inhibited cell apoptosis rate in Huh7 cells induced by LINC01503 overexpression were reversed by SCH772984 (both *P*<0.01) (Figure 6C, D). These results suggested that MAPK/ERK signal might be a pathway for LINC01503 to affect the biological behaviors of HCC cells.

## Discussion

In the last few decades, tremendous studies have been carried out to explore the pathogenicity and molecular mechanisms of HCC. However, the incidence and cancer-specific mortality still keep on increasing in many countries 17, 18. Recently, lncRNAs were reported to play an important part in various physio-pathological activities such as tumor pathogenesis 19-22. These studies offered us a hint that lncRNAs might be a kind of novel hallmark for diagnosis and new therapeutic targets of cancers. LINC01503 has been verified to promote the progress of esophageal squamous cell carcinoma, colorectal cancer, glioma, etc. 8-11. However, whether and how LINC01503 influences HCC still remain largely unknown. Firstly, we found that LINC01503 levels were significantly increased in HCC tissues in comparison with normal liver tissues by analyzing gene expression profiles from TCGA, which was further confirmed by the results of real-time PCR in clinical samples and HCC cell lines. Moreover, the LINC01503 expression correlated closely with high grade, high stage, large tumor size (T classification), as well as low overall survival rate in HCC patients. The LINC01503 expression was an independent risk factor for overall survival among HCC patients. The evidence above hinted that LINC01503 might be a promising prognostic biomarker for HCC.

But how do lncRNAs affect HCC progression? There are crucial evidences manifesting that lncRNAs deregulation might influence the proliferation, invasion, migration, apoptosis and metastasis of tumor 23-26. Consistent with the previous results 11, GO analysis indicated that high expression of LINC01503 was involved in some biological processes such as pro-proliferation and anti-apoptosis in HCC. In this research, the findings uncovered that silencing of LINC01503 could prevent HCC cell proliferation and promote cell apoptosis, while LINC01503 overexpression had the opposite effects. The upregulation and downregulation of LINC01503 in 2 kinds of HCC cells demonstrated that it might be a kind of carcinogenic gene accordantly. In addition, the nude mice were employed, and it was confirmed that LINC01503 is essential for liver tumor growth. Hence, LINC01503 might play a detrimental role upon the proliferation and progression of HCC. Though LINC01503 was reported to correlate with tumor metastasis and invasion in esophageal squamous cell carcinoma 8, we did not find significant relationship between tumor metastasis/invasion and LINC01503 in TCGA HCC samples by clinical correlation analysis and GSEA analysis.

Increasing reports have indicated that most HCC cases have genomic alterations in MAPK signaling pathway 27, 28. Interestingly, we found that MAPK signaling pathway is one of the LINC01503-related signaling pathways through KEGG analysis of the HCC samples in TCGA database. Hence, it is conceivably hypothesized that LINC01503 plays a role in HCC through the MAPK signaling pathway. MAPKs are serine/threonine protein kinases, which include ERK1/2, JNK, and P38 29. The activation of MAPK/ERK has been demonstrated to promote proliferation and inhibit apoptosis in multiple kinds of cancer, including HCC 14, 30. Our study verified that overexpression of LINC01503 could up-regulate the level of p-ERK, which could be down-regulated by silencing of LINC01503 in different HCC cells. Furthermore, the positive balance of proliferation/apoptosis induced by LINC01503 was reversed by ERK inhibitor. Nevertheless, significant changes of p-JNK and p-P38, which were also involved MAPK signaling pathways, were not observed, indicating LINC01503 might have no impact on these molecules in HCC. These results suggested that MAPK/ERK signaling pathway is essential for LINC01503 to promote proliferation and inhibit apoptosis in HCC. However, the exact sites mediating the association between LINC01503 and MAPK/ERK pathway were not illuminated. Hence, more detailed research should be conducted to entirely reveal the molecular cross-talk.

## Conclusion

In conclusion, our findings firstly demonstrated that LINC01503 was remarkably upregulated in hepatocellular carcinoma and served as an oncogene in HCC via activating MAPK/ERK pathway. This presented a novel insight that LINC01503 may become a potential biomarker and therapy target of HCC patients.

## Figures and Tables

**Table 1:** Characteristics of patients with hepatocellular carcinoma in TCGA.

Clinical characteristics		Total (374)	%
Age at diagnosis (y)	16-90	370	
Gender	Female	121	32.6
	Male	250	67.4
Grade	G1	55	15.0
	G2	177	48.4
	G3	122	33.3
	G4	12	3.3
Stage	Stage I	171	49.3
	Stage II	86	24.8
	Stage III	85	24.5
	Stage IV	5	1.4
T	T1	181	49.2
	T2	94	25.5
	T3	80	21.7
	T4	13	3.5
M	M0	266	98.5
	M1	4	1.5
N	N0	252	98.4
	N1	4	1.6

**Table 2:** The expression^a^ of LINC01503 related to clinicopathological features (logistic regression).

Clinical features	Total (N)	Odds ratio in the LINC01503 expression (95% CI)	*P*-Value
Age (age>50 *vs.* <=50)	370	1.00(0.61-1.65)	1.000
Gender (male *vs*. female)	371	0.69(0.44-1.06)	0.090
Grade (G4 *vs.* G1)	67	4.86(1.29-23.80)	**0.029**
Stage (II *vs.* I)	257	1.82(1.08-3.09)	**0.025**
(III *vs*. I)	256	1.78(1.06-3.03)	**0.031**
(IV *vs*. I)	176	5.50(0.79-108.81)	0.131
T (T2~4 *vs*. T1)	368	1.65(1.10-2.50)	**0.017**
M (M1 *vs*. M0)	270	3.05(0.38-62.00)	0.338
N (N1 *vs*. N0)	256	5943549(3.508989e-30-NA)	0.983

^a^ Categorical dependent variable, greater or less than the median expression level. Bold values indicate *P*<0.05. OR referred to odds ratio; CI was the abbreviation of confidence interval.

**Table 3:** Associations of overall survival with clinicopathological features in TCGA patients (Cox regression).

Clinicopathological features variable	Univariate analysis		Multivariate analysis	
	HR (95% CI)	*P*-Value	HR (95% CI)	*P*-Value
Age	1.01(0.99-1.02)	0.591		
Gender (male *vs.* female)	0.78(0.49-1.25)	0.301		
Grade	1.02(0.75-1.39)	0.914		
Stage	1.86(1.46-2.39)	**8.07E-07**	1.35(0.58-3.16)	0.488
T classification	1.80(1.43-2.27)	**4.73E-07**	1.36(0.62-2.98)	0.436
M classification	3.85(1.21-12.28)	**0.023**	0.86(0.23-3.13)	0.816
N classification	2.02(0.49-8.28)	0.328		
LINC01503 expression (high *vs.* low)	1.30(1.06-1.60)	**0.013**	1.26(1.01-1.57)	**0.046**

HR referred to hazard ratio; CI was the abbreviation of confidence interval. Bold values indicated *P*<0.05.

**Table 4:** Gene sets enriched in the high LINC01503 expression phenotype.

Gene set name	NES	NOM *P*-value	FDR *q*-value
POSITIVE_REGULATION_OF_CELL_PROLIFERATION	1.922	0.000	0.022
NEGATIVE_REGULATION_OF_APOPTOSIS	1.844	0.000	0.036
KEGG_MAPK_SIGNALING_PATHWAY	1.637	0.012	0.067

NES is the abbreviation of normalized enrichment scores. NOM, nominal; FDR is the abbreviation of false discovery rate. Gene sets with NOM *P*-value <0.05 and FDR *q*-value <0.25 were considered enriched significantly.

## Abbreviations

GO: Gene Ontology
GSEA: Gene Set Enrichment Analysis
HCC: hepatocellular carcinoma
HR: hazard ratio
KEGG: Kyoto Encyclopedia of Genes and Genomes
LIHC: liver hepatocellular carcinoma
NES: normalized enrichment scores
OR: odds ratio
OS: overall survival
RT-PCR: reverse transcription-PCR
TCGA: The Cancer Genome Atlas
